# The interaction of perceptual biases in bistable perception

**DOI:** 10.1038/srep42018

**Published:** 2017-02-06

**Authors:** Xue Zhang, Qian Xu, Yi Jiang, Ying Wang

**Affiliations:** 1 State Key Laboratory of Brain and Cognitive Science, CAS Center for Excellence in Brain Science and Intelligence Technology, Institute of Psychology, Chinese Academy of Sciences, 16 Lincui Road, Beijing 100101, P. R. China; 2 University of Chinese Academy of Sciences, 19A Yuquan Road, Beijing 100049, P. R. China

## Abstract

When viewing ambiguous stimuli, people tend to perceive some interpretations more frequently than others. Such perceptual biases impose various types of constraints on visual perception, and accordingly, have been assumed to serve distinct adaptive functions. Here we demonstrated the interaction of two functionally distinct biases in bistable biological motion perception, one regulating perception based on the statistics of the environment – the viewing-from-above (VFA) bias, and the other with the potential to reduce costly errors resulting from perceptual inference – the facing-the-viewer (FTV) bias. When compatible, the two biases reinforced each other to enhance the bias strength and induced less perceptual reversals relative to when they were in conflict. Whereas in the conflicting condition, the biases competed with each other, with the dominant percept varying with visual cues that modulate the two biases separately in opposite directions. Crucially, the way the two biases interact does not depend on the dominant bias at the individual level, and cannot be accounted for by a single bias alone. These findings provide compelling evidence that humans robustly integrate biases with different adaptive functions in visual perception. It may be evolutionarily advantageous to dynamically reweight diverse biases in the sensory context to resolve perceptual ambiguity.

When confronted with an ambiguous stimulus, the human brain can rapidly achieve an unambiguous interpretation, which is usually accompanied by biases^1^,^2^. This perceptual inference process is of great adaptive value, as it ensures the efficiency of perception and helps us overcome the processing limitations of the sensory system. Less tangibly, but perhaps more importantly, the biases that come with the inference process may also serve essential adaptive functions^3^,^4^. These biases impose constraints on perception by systematically enhancing our sensitivity to certain kinds of information. In particular, they are based on specific assumptions that arise at least from two sources, according to a theoretical framework for understanding the evolution of cognitive biases^4^.

The first set of biases is derived from prior knowledge about the environment. This sort of knowledge, represented as prior probability distributions in our brain as proposed by the Bayesian theory of human perception^5^,^6^,^7^, biases perception toward interpretations that match the statistics of the environment^8^,^9^,^10^,^11^. Consistent with this idea, people immediately perceive an ambiguous shape with shading at its lower part as a bump, assuming that the source of illumination is from above^12^,^13^. In the same vein, they tend to experience ambiguous lines^14^, surfaces^15^, Necker cubes^16^,^17^,^18^ or biological motions^19^ as viewing-from-above (VFA) at first glance, which is known as the VFA bias, due to the prior knowledge that objects most probably sit on surfaces below eye level.

Another category of perceptual biases is based on assumptions concerning the consequences of human behaviors. According to the error management theory, a bias leading to less costly errors will evolve^20^,^21^. Therefore, if missing a signal would cost higher than a false-alarm, a bias towards such signal can arise. This can partially explain the enhanced sensitivity to information that is potentially dangerous^22^,^23^ as well as the asymmetry in the perception of approaching and receding stimuli^24^,^25^,^26^,^27^. For example, people are more likely to perceive ambiguous moving patterns as approaching motion rather than receding motion^28^. Similarly, they perceive an ambiguous human walker as facing-the-viewer (FTV) more frequently than the opposite, i.e., away-from-the-viewer^29^,^30^,^31^,^32^, exhibiting the FTV bias, and have superior sensitivity to the FTV walkers^33^,^34^,^35^.

The two types of perceptual biases, namely, the “statistics-based biases” and the “error management biases”, represent different facets of the perceptual inference process. Yet it remains unclear whether our brain combines such discrepant biases to make sense of the sensory world. When two biases from different categories coexist, and each of the biases alone can enable us to resolve the ambiguity in the sensory input, does the brain rely on one bias while suppressing the other, or allow the two biases to interact? Moreover, considering that perception hinges on not only biases but also the sensory information, it raises the question of whether visual cues that alter the ambiguity of the stimulus could modulate the interaction of the biases.

To address these issues, we examined the FTV bias and the VFA bias, given that they belong to the two aforementioned categories respectively and can arise within a single scenario, i.e., biological motion perception. We adopted orthographically projected point-light-walker (PLW) stimuli^36^ and manipulated the angular deviation from the horizontal plane (Fig. 1A). Without explicit depth cues, these stimuli were ambiguous in terms of orientation-in-depth (toward or away from the viewer) and viewpoint (from above or below). We generated two kinds of ambiguous PLW stimuli, each having two possible interpretations (Fig. 1B). One stimulus could be perceived as viewed from above and walking toward viewer (Above-Toward) or viewed from below and walking away from viewer (Below-Away). We defined it as the consistent condition where the two percepts were either congruent (VFA+ FTV+ ) or incongruent (VFA−FTV−) with both biases. The other stimulus could be perceived as viewed from above while walking away from viewer (Above-Away) or viewed from below while walking toward viewer (Below-Toward). We defined it as the conflicting condition in which one percept was compatible with the VFA bias and opposed to the FTV bias (VFA+ FTV−) while the other was consistent with the FTV bias but against the VFA bias (FTV+ VFA−).

If the brain simultaneously takes into account both the VFA and the FTV assumptions, rather than relying on a single one, as we expected, the overall strength of bias should be modulated by the interaction mode. Particularly, there should be stronger bias in the consistent than in the conflicting condition. Moreover, we predicted that the two biases might compete with each other when they were in conflict, and the competition would depend on visual cues that modulate the ambiguity of the stimulus. To examine these assumptions, we varied the camera angle between 45° and 5° with respect to the horizontal plane using the orthogonal projection method (Fig. 1A). This angular cue was unlikely to directly benefit the resolution of ambiguity, as it did not provide any information about the viewpoint (above/below) or walking direction (toward/away). However, it was possible to modulate the weights of the two biases in the perceptual inference process by amplifying the ambiguity related to the VFA assumption (at relatively large angle) or the FTV assumption (at relatively small angle).

## Results

### Experiment 1

We first set up a pilot experiment to check whether observers could experience the two possible interpretations of the PLW stimuli with large angle of elevation/depression (45°), because no previous studies have examined the perception of such stimuli. Another aim of this experiment was to explore whether interaction modes (consistent and conflicting) of the biases could influence the dynamics of perceptual reversals. Observers were asked to watch the PLW stimuli and report the switches of perception in the consistent condition and the conflicting condition respectively. As shown in Fig. 2A, observers did experience reversals of perception in both conditions during the 3 min trials, although there were large individual variations in switch rates. More importantly, fewer perceptual reversals were observed in the consistent condition than in the conflicting conditions (Fig. 2B; *t*(7) = −2.84, *p* = 0.03), suggesting that the consistency between the two biases enhances the stability of bistable perception.

### Experiment 2

We further investigated the interaction of the two biases and the potential influence of camera angle cue when the angular deviation from the horizontal plane was relatively large (35°, 20°), in Experiment 2a, and relatively small (15°, 10°, 5°), in Experiment 2b.

In Experiment 2a, the mean proportion of Above_Toward responses (VFA+ FT+) in the consistent condition and the mean proportion of Above_Away responses (VFA+ FTV−) in the conflicting condition were both significantly higher than 50% (Fig. 3A, left; one sample T-test: Consistent: *t*(9) = 27.64, *p* < 0.001; Conflicting: *t*(9) = 6.69, *p* < 0.001), suggesting that the VFA bias dominates perception when the angle of elevation/depression is large. Furthermore, we used the proportion of the dominant percept for each experimental condition, i.e., the percept whose proportion was larger than 0.5 (Fig. 3B, left; Above_Toward for the consistent condition vs. Above_Away for the conflicting condition) as a measure of the strength of bias, and submitted the data to a 2 (Consistent vs. Conflicting) × 2 (35° vs. 20°) repeated measures ANOVA. Neither the main effect of the two factors (interaction mode: (*F*(1, 9) = 1.28, *p* = 0.29; angular deviation: *F*(1, 9) = 1.79, *p* = 0.21) nor their interaction (*F*(1, 9) = 1.71, *p* = 0.22; Fig. 3B left) was significant, indicating that large angular deviation from the horizontal plane prevents the VFA assumption from interacting with the FTV assumption.

In Experiment 2b, the mean proportion of Above_Toward responses in the consistent condition was still significantly higher than 50% (*t*(9) = 9.54, *p* < 0.001; Fig. 3A, right). Whereas in the conflicting condition, the proportion of Above_Away responses became significantly lower than 50% (*t*(9) = 3.00, *p* = 0.02; Fig. 3A right), suggesting that the VFA assumption gives way to the FTV assumption. A two-way repeated measures ANOVA on the proportion of the dominant percept (Fig. 3B, right; Above_Toward for the consistent condition vs. Below_Toward for the conflicting condition) revealed a significant main effect of interaction mode (*F*(1, 9) = 9.23, *p* = 0.01) and a significant interaction between interaction mode and angular deviation (*F*(2, 18) = 5.64, *p* = 0.01; Fig. 3B right). Specifically, the strength of bias increased as the angular deviation became smaller (15° vs. 10°: *t*(9) = −2.43, *p* = 0.04; 10° vs. 5°: *t*(9) = −1.1, *p* = 0.30; Fig. 3B right), only in the conflicting condition.

Taken together, the results of Experiment 2a and 2b provide clear evidence for the interaction of the VFA and the FTV biases. On the one hand, the general strength of bias was stronger in the consistent condition than in the conflicting condition, especially when the angular deviation was small, suggesting that the brain combines the two biases to constrain perception. On the other hand, the dominant percept reversed between large and small angles in the conflicting condition, indicating that rivalry occurs between the two biases.

### Experiment 3

Experiment 2 revealed the role of sensory information in modulating the rivalry of biases, by demonstrating the differences between the large angle and small angle conditions. To further elucidate the influence of camera angle on the interaction of the VFA and the FTV biases, we conducted Experiment 3, in which we systematically varied the angle of deviation from the horizontal plane (25°, 20°, 15°, 10°, and 5°) as a within-subjects factor.

The proportion of Above_Toward responses in the consistent condition was again higher than 50% for all deviation angles (*p* < 0.001; Fig. 4A). By contrast, the proportion of Above_Away responses in the conflicting condition was higher than 50% only at 25°(*t*(15) = 2.01, *p* = 0.06), with the pattern reversed when the angle of deviation decreased to 5° (*t*(15) = 2.19, *p* = 0.04), which was consistent with the findings from Experiment 2. Moreover, a 2 (interaction mode) × 5 (angular deviation) repeated measures ANOVA on the proportion of the dominant percept (Fig. 4B) showed significant main effects of interaction mode (*F*(1, 15) = 17.38, *p* = 0.001) and angular deviation (*F*(4, 60) = 20.89, *p* < 0.001), and a significant interaction between the two factors (*F*(4, 60) = 5.94, *p* < 0.001). Specifically, the weakest bias came with the angle of 10°, and the strength of bias became stronger as the angle of deviation became either larger (15° vs. 10°: *t*(15) = 2.52, *p* = 0.02; 20° vs. 10°: *t*(15) = 3.31, *p* < 0.01; 25° vs. 10°: *t*(15) = 4.12, *p* < 0.001) or smaller (5° vs. 10°: *t*(15) = 2.94, *p* = 0.01), though in those two situations perception was actually biased toward opposite directions. Such pattern was only observed in the conflicting condition, suggesting that angular deviation from the horizontal plane modulates the rivalry between the two biases.

Interestingly, further examination of individual data revealed that there were large individual differences regarding the dominant percept (Fig. 4C). Some observers consistently manifested a preference for the VFA assumption over the FTV assumption (VFA-dominated), while others exhibited the opposite pattern (FTV-dominated) or a relatively neutral pattern (no apparent preference). Despite such dramatic difference, almost all observers showed the tendency that the proportion of the Above_Away responses decreased gradually with the angle of deviation in the conflicting condition, whereas the proportion of Above_Toward responses was relatively stable across different angles. In addition, it is noteworthy that there is no significant correlation between the VFA percepts in the two conditions (i.e., ‘Above_Away’ responses in the conflicting condition and ‘Above_Toward’ responses in the consistent condition) at each camera angle (Fig. 4C, lower panels; 25°: r = 0.31, *p* = 0.24; 20°: r = 0.27, *p* = 0.31; 15°: r = 0.27, *p* = 0.31; 10°: r = 0.29, *p* = 0.27; 5°: r = −0.14, *p* = 0.60). These results suggest that the observations from the current study are not likely due to the effect merely related to a single bias, because a single-bias-dominant effect would lead to strong correlations between the two conditions in terms of VFA percept or FTV percept.

To sum up, results of Experiment 3 demonstrate that angular deviation modulates the relative weight of the two biases independent of the weight of any single bias at the individual level, which highlights a mechanism underlying visual perception for dynamically reweighting biases with different functions according to the sensory input.

## Discussion

The current study investigated the interaction of the VFA bias and the FTV bias, two perceptual biases that have been assumed to serve distinct adaptive functions, in bistable visual perception. We found that when compatible, the two biases produced a robust bias effect, with the strength of bias being stronger (Experiment 2 and Experiment 3) and the perception being more stable (Experiment 1) relative to when the two biases were in conflict. On the other hand, in the conflicting condition, the two biases competed with each other, with their relative strength modulated by the angular deviation from the horizontal plane (Experiment 2 and 3). These findings provide compelling evidence that the human brain spontaneously integrates biases driven by different evolutionary forces to resolve visual ambiguity, and suggest that the interaction of the biases can be modulated by the sensory cues.

These results provide valuable clues about how biases with distinct adaptive goals constrain perception in situations with different degrees of complexity. In a relatively simple situation where a possible percept can meet multiple adaptive goals (e.g., the consistent condition), such percept is undoubtedly the best and simplest solution. Nevertheless, in a situation where no such perfect solution exists (e.g., the conflicting condition), the brain may evaluate the nature of the biases as well as information provided by the sensory input to resolve the ambiguity.

Specifically, we found the interaction of the biases hinged on the camera angle cue only when the two biases were in conflict: at the group level, the VFA bias trumped the competition at relatively large angle, while the FTV bias gained dominance at relatively small angle. This observation can be accounted for by the functional distinction between the two biases. From an evolutionary perspective, the VFA bias probably serves to constrain perceptual inference by applying prior knowledge about the physical environment^8^,^9^,^10^,^11^, whereas the FTV bias may exist to reduce the likelihood that one would miss the potential threat linked with an approaching creature^37^,^38^,^39^,^40^,^41^,^42^,^43^. This difference allows the weights of the two biases to change in opposite directions with the camera angle cue. In particular, the larger the angular deviation was, the more imperative solving the ambiguity regarding the viewpoint (from above or from below) became, and then a greater weight would be given to the VFA bias. Conversely, smaller deviation angles indicated a larger possibility that an approaching walker would interact with the viewer, thereby promoting the necessity to deal with the ambiguity regarding orientation-in-depth and requiring the brain to give more priority to the FTV bias. This twofold effect may give rise to the reweighting of the two biases we observed at the group level. Crucially, this pattern was consistent across observers, no matter which bias dominated perception at the individual level, and no significant correlation was observed between the two conditions in terms of the VFA percept (or the FTV percept), further excluding the possibility that the competition of the two biases is governed by any single bias. Rather, our results support the existence of a mechanism underlying perceptual inference for adaptively reweighting diverse heuristic assumptions in accordance with the sensory input.

The weight of a bias varies with the visual cue is consistent with the Bayesian theory of perception^5^,^6^,^7^. In a computational model that has been proposed to account for the interaction of visual constraints, the relative weight of two competing priors that are both based on environmental statistics may change as a function of the reliability of their corresponding visual cues^15^. It is plausible to assume that the interaction of the VFA bias and the FTV bias also follows the rules of Bayesian inference, with the general bias strength predicted by visual cues that can modulate both biases simultaneously, such as the camera angle. However, to incorporate this assumption, the current theoretical and computational frameworks for the interaction of prior constraints need to be extended to include biases that arise from sources beyond the environmental statistics, such as the FTV bias or the biases concerning the asymmetry in the perception of potentially threatening and neutral stimuli^24^,^25^,^26^,^27^,^37^,^38^,^39^,^40^. One major challenge posed by these “error-management-biases” is that they may have more complex sources than the “statistics-based priors”. For example, several studies argue for the important role of physical cues, such as surface form or local motion, in the FTV bias^44^,^45^,^46^. In addition, our recent study reveals a significant and specific contribution of genetic factors to the individual differences in the FTV bias^47^. Future studies should consider the potential factors contributing to the FTV bias or other “error management biases”, and illuminate how these factors modulate the interaction between these biases and the “statistics-based biases” using psychophysical and modeling methods.

In conclusion, the current study demonstrates the interaction of two biases shaped by distinct adaptive forces (i.e., the VFA bias and the FTV bias) in bistable visual perception. The two biases cooperate to strengthen the percept compatible with both biases, whereas rival for dominance in a stimulus-sensitive manner when in conflict. These findings shed new lights on how the human brain combines biases serving various adaptive functions to gain optimal perception, and emphasize the need to understand the functioning and interaction of perceptual biases in the sensory context.

## Methods

### Participants

The study was carried out in accordance with procedures and protocols approved by the institutional review board of the Institute of Psychology, Chinese Academy of Sciences. A total of 47 (23 female) undergraduate and graduate students participated in the study, 8 for Experiment 1, 10 for Experiment 2a, 13 for Experiment 2b, and 16 for Experiment 3. Three participants from Experiment 2b were excluded from data analysis due to low response accuracy (see details in Data Analysis). All participants had normal or corrected-to-normal vision, and were naïve to the purpose of the experiments. They gave written informed consent and were paid for their participation.

### Stimuli

**Figure 1:**
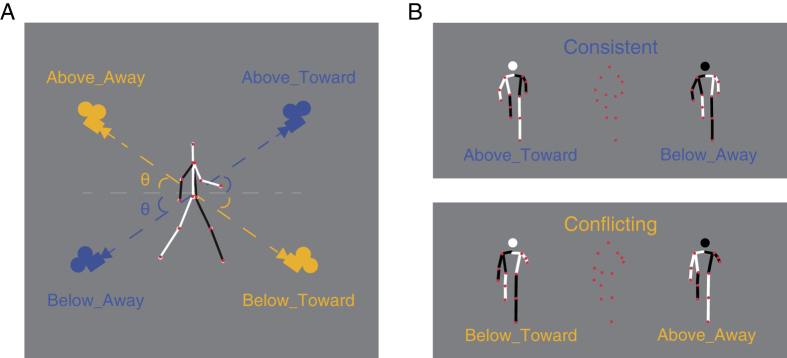
Illustrations of bistable point-light walker (PLW) stimuli and the possible percepts in the consistent and the conflicting experimental conditions. The lines connecting the dots (displayed for illustration only) illustrate the depth information: white lines represent the body parts closer to the viewer, and black lines represent parts that are farther in depth. (**A**) Viewpoints of the two possible percepts for the consistent condition, indicated by blue cameras; and for the conflicting condition, indicated by orange cameras. θ represents the angle of deviation from the horizontal plane, which ranges from 45° to 5° and can be considered as either the angle of elevation or the angle of depression for each stimulus, depending on which percept is referred to. (**B**) Orthogonally projected PLW stimuli (central) and their corresponding percepts (left and right).

**Figure 2:**
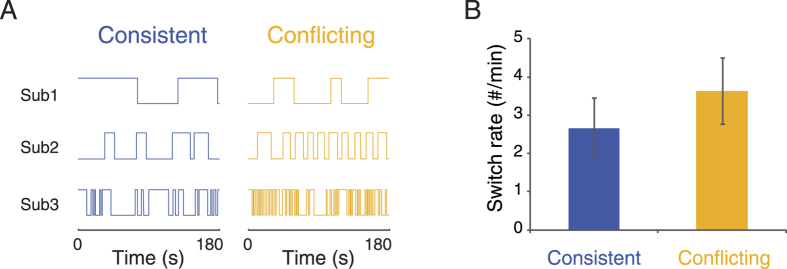
Results of Experiment 1. (**A**) Perceptual reversals as a function of time in the consistent and the conflicting conditions. The panels from top to bottom display sample trials from three observers with slow, medium and fast switch rates, respectively. (**B**) Mean switch rates for the consistent and the conflicting conditions. Error bars denote ± SEM.

**Figure 3:**
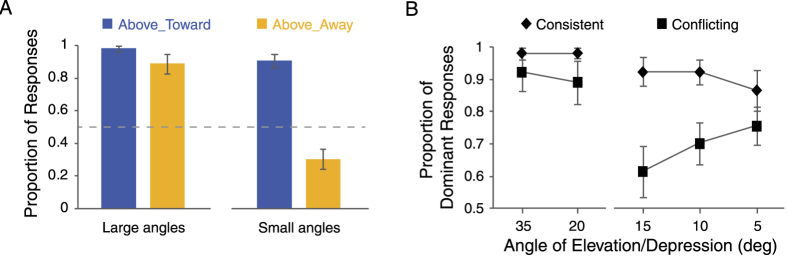
Results of Experiment 2a (angular deviations: 35°, 20°; large angles) and Experiment 2b (angular deviations: 15°, 10°, 5°; small angles). (**A**) Mean proportions of Above_Toward responses in the consistent condition and Above_Away responses in the conflicting condition for Experiment 2a (Large angles) and 2b (Small angles). (**B**) Mean proportions of the dominant responses in the consistent and the conflicting conditions respectively at each angular deviation. Error bars denote ± SEM.

**Figure 4:**
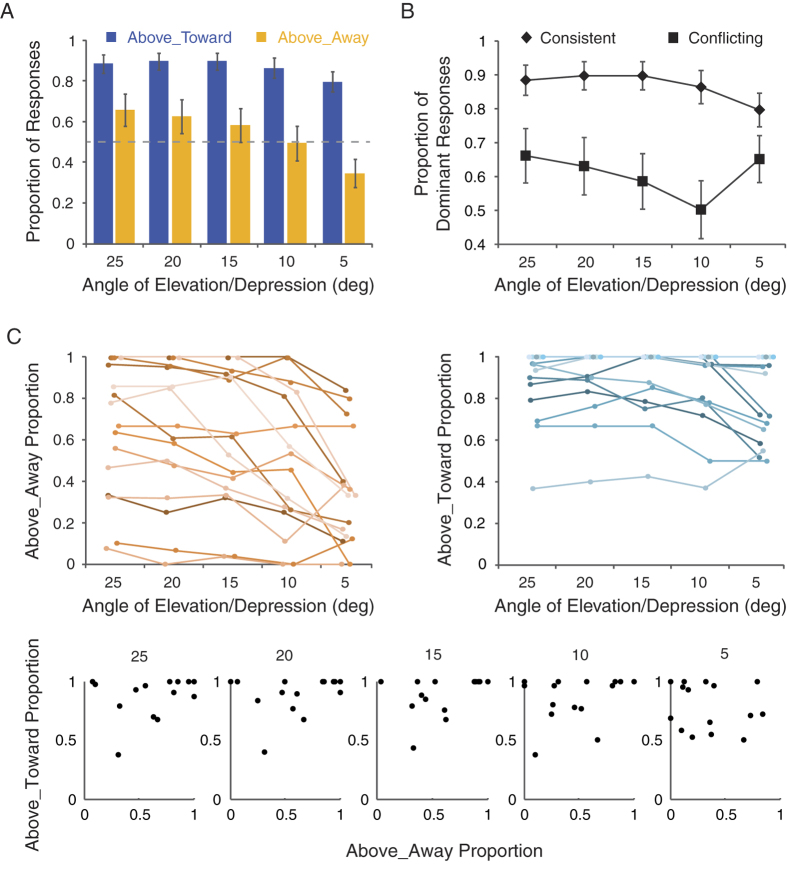
Results of Experiment 3 (angular deviations: 25°, 20°, 15°, 10°, 5°). (**A**) Mean proportions of Above_Toward responses in the consistent condition and Above_Away responses in the conflicting condition as a function of angular deviation. (**B**) Mean proportions of the dominant responses along 5 angles of deviation in the consistent and the conflicting conditions. (**C**) Proportions of Above_Away responses in the conflicting conditions (upper-left panel) and proportions of Above_Toward responses in the consistent conditions (upper-right panel) for individual observers, as well as the correlation between them at each camera angle (lower panels). Error bars denote ± SEM.

Stimuli were generated and displayed using MATLAB together with the Psychophysics Toolbox extensions^48^,^49^. The point-light walker (PLW) consists of 13 white dots placed on the major joints and the head of a human walker, and can be perceived as either facing toward or away from the observer^50^. We rotated the walkers with respect to the horizontal plane (by 45° in Experiment 1; by 35° and 20° in Experiment 2a; 15°, 10° and 5° in Experiment 2b; and 25°, 20° 15°, 10°, 5° in Experiment 3) to change the angle of deviation from horizontal. Note that for each stimulus, the angle of deviation could be considered as either the angle of elevation (for one possible percept) or the angle of depression (for the other percept), resulting in ambiguity regarding the observer’s viewpoint (from above or from below). By this means, we generated two kinds of ambiguous PLW stimuli belonging to the consistent and the conflicting experimental conditions respectively (Fig. 1).

### Procedure

Participants sat in front of a 19-inch cathode ray tube display (1280 × 1024, 60 Hz) with a viewing distance of 60 cm. Each trial began with a PLW stimulus displayed with a white fixation cross at the center of the screen on gray background. The stimuli (ranging from 5.7 to 6.7 degrees in height, dependent on the angular deviation from the horizontal plane) were presented in one of three horizontal directions: directly toward the viewer (0°), 45° left and 45° right.

In Experiment 1, the PLW stimulus was displayed for 3 min in each trial. Observers were asked to report the walking direction of the PLW when it switched between facing toward viewer and away from viewer by pressing the corresponding keys. Before the formal test, observers received practice trials to get familiar with all the stimuli. The formal test consisted of 6 trials in total, with an equal number of trials in the consistent and the conflicting conditions. The order of conditions was counterbalanced across observers. To reduce the potential influence from the previous trial, observers were required to take a compulsive break for 1 minute after each trial.

In Experiments 2 and 3, the PLW stimulus was displayed for 1000 ms in each trial. Observers were required to judge (1) the walking direction of the PLW (e.g., toward the viewer and to the observer’s left) and (2) the viewpoint with respect to the horizontal plane (viewing from above or from below) successively by pressing the corresponding keys. There were 120 trials in Experiment 2a, 180 trials in Experiment 2b and 300 trials in Experiment 3, with 30 trials for each experimental condition (consistent or conflicting condition combined with each angular deviation from the horizontal plane). Within each experiment, all trials were run in random order. The observers received a practice session before the formal test, and took compulsive breaks every 20 trials during the experiment.

### Data analysis

For Experiment 1, percepts lasting for less than two seconds were considered unstable and excluded from further analysis. For Experiments 2 and 3, we only included the correct trials, i.e., trials with responses matching any of the two possible interpretations of the ambiguous stimulus. In Experiment 2b, three subjects whose mean accuracy was below the chance level computed based on a binomial distribution with a probability threshold at 0.95 were excluded from analysis. For all experiments, data of the three horizontal directions were combined for formal analyses, since preliminary analyses revealed no significant main effect of horizontal direction.

## Additional Information

**How to cite this article**: Zhang, X. *et al*. The interaction of perceptual biases in bistable perception. *Sci. Rep.* **7**, 42018; doi: 10.1038/srep42018 (2017).

**Publisher's note:** Springer Nature remains neutral with regard to jurisdictional claims in published maps and institutional affiliations.
